# Nix Plays a Neuroprotective Role in Early Brain Injury After Experimental Subarachnoid Hemorrhage in Rats

**DOI:** 10.3389/fnins.2020.00245

**Published:** 2020-03-24

**Authors:** Juyi Zhang, Guiqiang Yuan, Tianyu Liang, Pengjie Pan, Xiang Li, Haiying Li, Haitao Shen, Zhong Wang, Gang Chen

**Affiliations:** Department of Neurosurgery & Brain and Nerve Research Laboratory, The First Affiliated Hospital of Soochow University, Suzhou, China

**Keywords:** Nix, subarachnoid hemorrhage, early brain injury, neuroprotection, TOMM20

## Abstract

Nix is located in the outer membrane of mitochondria, mediates mitochondrial fission and implicated in many neurological diseases. However, the association between Nix and subarachnoid hemorrhage (SAH) has not previously been reported. Therefore, the present study was designed to evaluate the expression of Nix and its role in early brain injury (EBI) after SAH. Adult male Sprague-Dawley (SD) rats were randomly assigned to various time points for investigation after SAH. A rat model of SAH was induced by injecting 0.3 ml of autologous non-heparinized arterial blood into the prechiasmatic cistern. The expression of Nix was investigated by Western blot and immunohistochemistry. Next, Nix-specific overexpression plasmids and small interfering RNAs (siRNAs) were separately administered. Western blot, neurological scoring, Morris water maze, terminal deoxynucleotidyl transferase-mediated dUTP nick end labeling (TUNEL) staining and fluoro-jade B (FJB) staining were performed to evaluate the role of Nix in EBI following SAH. We found that Nix was expressed in neurons and its expression level in the SAH groups was higher than that in the Sham group, which peaked at 24 h after SAH. Overexpression of Nix following SAH significantly decreased the expression of translocase of outer mitochondrial membrane 20 (TOMM20, a marker of mitochondria), ameliorated neurological/cognitive deficits induced by SAH, and reduced the total number of apoptotic/neurodegenerative cells, whereas siRNA knockdown of Nix yielded opposite effects. Taken together, our findings demonstrated that the expression of Nix is increased in neurons after experimental SAH in rats, and may play a neuroprotective role in EBI following SAH.

## Introduction

Spontaneous subarachnoid hemorrhage (SAH) is a cerebrovascular disease with high disability and mortality rates. Rupture aneurysm is the main cause of SAH, accounting for about 85% of all spontaneous SAH (Ji and Chen, 2016). Although great progress has been made in current treatment of intracranial aneurysms, including microsurgery and endovascular embolization, it is reported that the disability and mortality rates of SAH have still not significantly decreased. The mortality rate of SAH is between 25 and 35% in high-income countries, and is as high as 48% in low-income countries (Pluta et al., 2009). Early brain injury (EBI) is an important factor leading to the deterioration of SAH patients. The occurrence of EBI after SAH is a complex pathophysiological process, and its mechanisms may be related to autophagy, apoptosis, inflammation, destruction of the blood-brain barrier (BBB), cytotoxic brain edema and oxidative stress (Fujii et al., 2013). Therefore, there is an urgent need for continued scientific investigations of novel approaches for preventing and treating EBI after SAH.

Autophagy involves the identification of cellular proteins and organelles for degradation that are then wrapped by double layered membranous structures that are detached from the ribosome-free attachment region of the rough-surface endoplasmic reticulum (ER) to form autophagosomes; subsequently, autophagosomes are further degraded after being fused with lysosomes, to meet the metabolic demands of cells and to regenerate organelles as needed (Nah et al., 2015). Studies have shown that mitochondrial dysfunction affects a series of intracellular biological processes that are involved in the process of EBI after SAH, including oxidative damage, calcium homeostasis disorder, and the collapse of ATP synthesis (Li et al., 2018). Mitophagy is the process in which injured or unwanted mitochondria are selectively cleared via autophagy, thereby maintaining the homeostasis of cells (Liu et al., 2014). Taken together, mitophagy may play an important role during EBI after SAH.

Nix, also known as B-cell lymphoma 2 (Bcl-2)-interacting protein 3 like (Bnip3L), was originally thought to belong to the Bcl-2 family and BH3-only pro-apoptotic proteins, but its ability to induce apoptosis was found to be weak (Sandoval et al., 2008; Zhang and Ney, 2009). With the development of further research, it has been found that Nix is different from other typical BH-3-only proteins and that pro-apoptosis may not be its main function (Mellor and Harris, 2007; Ding et al., 2010). Nix was first described as a mitophagic receptor when it was discovered to be involved in the procedural clearance of mitochondria during reticulocyte maturation (Ney, 2015). In recent years, it has been considered that the mechanisms of EBI after SAH are complex, which are the result of a combination of various factors. Although recent studies have suggested that autophagy is activated after SAH and can ameliorate EBI, the specific mechanism underlying this process remains unknown (Li et al., 2014). As mitochondria are the most important organelles for sources of intracellular energy, mitochondrial dysfunction plays a significant role in EBI after SAH (Wang et al., 2017; Zhou et al., 2017). Mitophagy clears impaired mitochondria in cells. Hence, discovery of the key factors that regulate the occurrence of mitophagy after SAH may reverse brain injury caused by mitochondrial dysfunction (Chen et al., 2014). Nix is located on the outer mitochondrial membrane and has been demonstrated to be a vital protein for regulating mitophagy. Moreover, Nix has been shown to be involved in the pathophysiological processes of various central nervous system (CNS) diseases, including cerebral ischemia-reperfusion (I/R) injury, intracranial hemorrhage, spinal cord injury and neurodegenerative diseases; however, the specific mechanisms of Nix in the pathophysiology of these CNS diseases remain unclear (Rui et al., 2013; Yu et al., 2013; Park et al., 2017; Yuan et al., 2017).

In the present study, we determined the expression of Nix after SAH, and further investigated the role of Nix in EBI after SAH. Our findings elucidating the role of Nix in EBI after SAH may provide a novel approach of thought for the treatment of SAH patients.

## Materials and Methods

### Animals

Adult male Sprague-Dawley (SD) rats weighing between 300 and 350 g were purchased from the Animal Center of the Chinese Academy of Sciences (Shanghai, China). All of the procedures were approved by the Institutional Animal Care Committee of the First Affiliated Hospital of Soochow University and were in accordance with the guidelines of the National Institutes of Health on the care and use of animals. All of the rats were housed in temperature- and humidity-controlled animal quarters with a 12 h light/dark cycle and free access to food and water.

### Experimental Design

In the first experiment, adult male SD rats were randomly assigned to eight groups of 12 rats each, as follow: a Sham group and seven SAH groups arranged by time: at 3, 6, 12, 24, 48 and 72 h, as well as 7 days, after SAH. All of the rats in the experiment were sacrificed at the matching time point after SAH. The brain tissue of six rats in each group was harvested for Western blotting and immunofluorescence analysis to investigate the expression of Nix. The experimental results were statistically analyzed.

**FIGURE 1:**
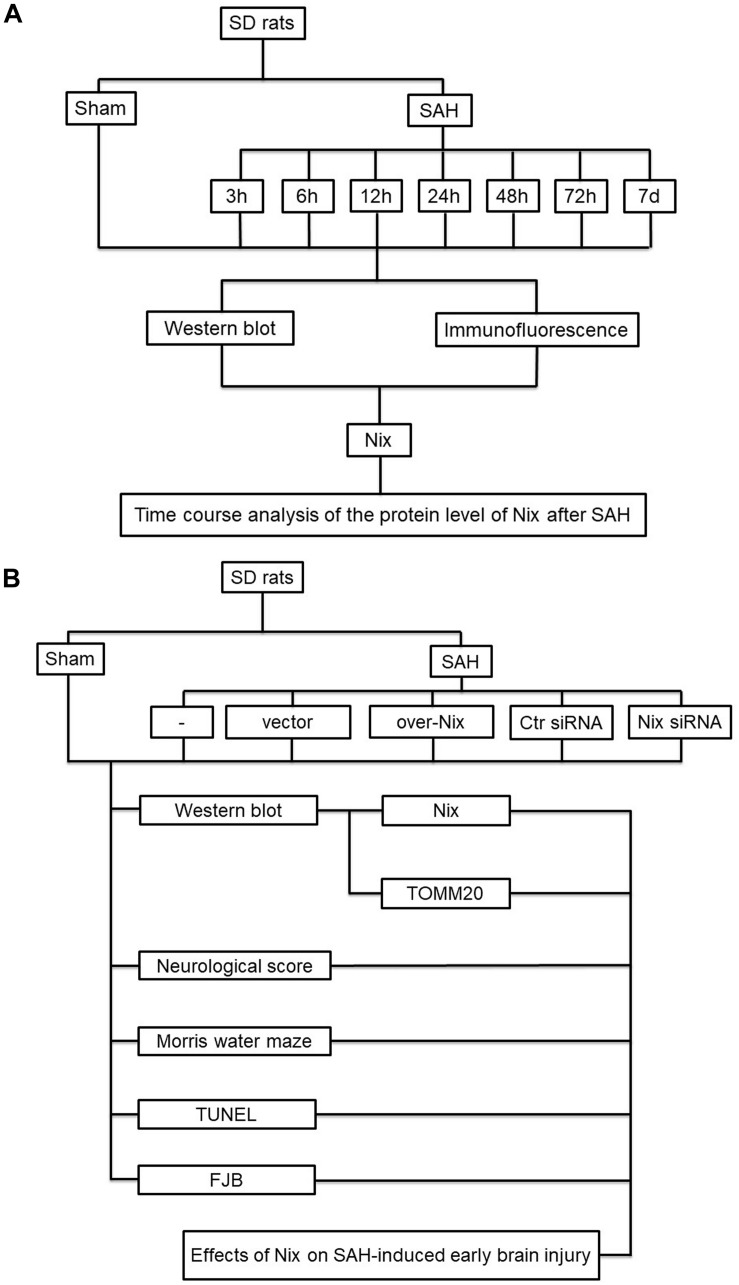
Experiment design. **(A)** The first part of the study was designed to evaluate the expression of Nix at different time points following SAH. **(B)** The second part of study investigated the effect of Nix on SAH-induced EBI.

In the second experiment, healthy adult male Sprague-Dawley rats were randomly divided into six groups of 18 rats each, as follow: Sham group, SAH group, SAH + vector (Vec) group, SAH + over-Nix group, SAH + Negative Control-siRNA (Ctr siRNA) group, and SAH + Nix siRNA group. In each group, the brain tissue of six rats in each group was harvested for Western blotting analysis to measure the expression levels of Nix and the mitochondrial marker protein, TOMM20. Another six rats were sacrificed for terminal deoxynucleotidyl transferase-mediated dUTP nick end labeling (TUNEL) staining and fluoro-jade B (FJB) staining to evaluate the neuronal apoptosis and neurodegeneration. The last six rats in each group were used for neurological scoring and assessment of spatial learning and cognitive capacity, which were tested using the Morris water maze (MWM; Figure 1).

### SAH Model

The prechiasmatic-cistern single-injection SAH model was performed as previously reported (Yuan et al., 2019). The rats were weighed and anesthetized by intraabdominal injection with 4% chloral hydrate at a dose of 10 ml/kg. After being fixed in the stereotaxic apparatus (Anhui Zhenghua Biological Equipment Co. Ltd., China), a circular bone window about 1–2 mm in diameter was drilled 7.5 mm anterior to bregma and 3.0 mm lateral to the midline by dental electric drilling. If needed, bone wax was used to prevent the loss of blood and cerebrospinal fluid (CSF) from the midline vessels when drilling the cranium. At an angle of 45° to the sagittal plane, the needle of a syringe was inserted 2–3 mm anterior to the chiasma (about 10–12 mm from the brain surface), and was retracted 0.5 mm. Then, 0.3 ml of autologous non-anticoagulant artery blood collected by cardiac puncture was injected into the prechiasmatic cistern for 20 s with a syringe pump under aseptic technique. Similarly, the 0.3 ml of saline was injected in the Sham group rats. SAH model rats may have had a heartbeat and spontaneous apnea, and some rats were able to restore their normal circulatory and respiratory function after being rescued by extrathoracic compression.

**FIGURE 2:**
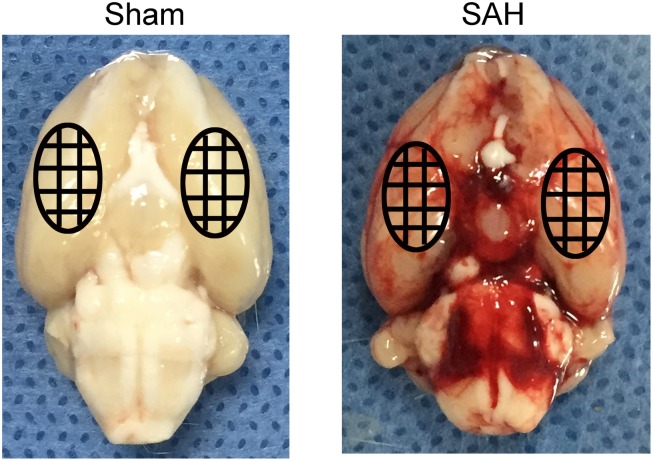
Representative images of whole brain from a Sham rat and a SAH model rat.

After the operations, the rats were returned to their cages and injected subcutaneously with 10 ml of saline to prevent postoperative dehydration, and the room temperature was kept at 23 ± 1°C. Vital signs were monitored continuously and the rectal temperature was kept at 37 ± 0.5°C (Figure 2). The severity of SAH was blindly calculated by a rating scale (Sugawara et al., 2008). After the rat was euthanized, we imaged that the base of its brain was divided into six parts. Each part had a score (0–3), which is corresponded to the amount of the blood over it. The scale’s maximum was 18. Rats with SAH grading scores < 8 were excluded and replaced from this study.

### Antibodies and Reagents

Anti-Nix antibody (12396S) was purchased from Cell Signaling Technology (United States). Anti-NeuN antibody-Neuronal cell marker (ab104224), anti-TOMM20 antibody (ab56783), and anti-β-tubulin antibody (ab179513) were purchased from Abcam (United States). Goat anti-rabbit IgG-HRP (sc-2004) and goat anti-mouse IgG-HRP (sc-2005) were purchased from Santa Cruz Biotechnology (United States). Alexa Fluor-488 donkey anti-rabbit IgG antibody (A21206) and Alexa Fluor-555 donkey anti-mouse IgG antibody (A31570) were purchased from Invitrogen (United States). Nix overexpression plasmid and siRNA were purchased from GenScript (China).

### Drug Administrations

Transfection was conducted as described previously (Dou et al., 2017; Shen et al., 2017). Briefly, 5 μg of Nix overexpression plasmid or vector plasmid was dissolved in 5 μl of endotoxin-free water, which was then added to 10 μl of *in vivo* DNA transfer reagent, which was mixed for 15 min at room temperature. In addition, 500 pmol of Nix siRNA or control siRNA was dissolved in 5 μl deionized water, and was then added to 10 μl of *in vivo* RNA transfection reagent, which was mixed for 15 min at room temperature. Finally, the mixture was injected into the lateral ventricle via stereotaxic coordinates from a rat brain atlas at 24 h before the SAH model was performed.

### Intracerebroventricular Injections

SD rats were weighed and anesthetized by intraperitoneal injection with 4% chloral hydrate at a dose of 10 ml/kg. After successful anesthesia, the rats were fixed on the stereotactic instrument, the scalp was cut along the median sagittal line, and the muscles and periosteum were carefully separated and expose to the skull. Then, a small burr hole was drilled into the skull 1.5 mm posterior to bregma and 1.0 mm lateral to the midline over the left hemisphere (Zhang et al., 2019). The needle of a 25 μl Hamilton microsyringe was slowly pierced through the hole and inserted into the left ventricle at 4.0 mm below the dural surface (Meng et al., 2018). The reagent was slowly injected into the left ventricle at a rate of 0.5 μl/min.

### Western Blot

Temporal-floor brain tissue of each rat was collected and homogenized in lysis buffer (Beyotime Institute of Biotechnology, China). The tissue homogenate was collected into a centrifuge tube, and then centrifuged at a speed of 12,000 rpm/min for 10 min at 4°C. Protein concentrations of the supernatants were measured through a bicinchoninic acid assay (BCA, Beyotime Institute of Biotechnology). Afterward, each protein sample (30 μg/lane) was loaded on a 12% SDS-PAGE gel, separated, and then electrophoretically transferred to a polyvinylidene difluoride (PVDF) membrane (Millipore Corporation, Billerica, MA, United States), which was subsequently blocked with 5% skim milk for 60 min at room temperature and then incubated with a primary antibody overnight at 4°C. The diluted horseradish peroxidase (HRP)-labeled secondary antibody was incubated for about 1 h at room temperature and then washed three times with PBST (PBS + 0.1% Tween 20). Finally, an enhanced chemiluminescence (ECL) kit was used for visualization of bands that were then analyzed via ImageJ software. Relative quantification of proteins was evaluated through normalizing to levels of the corresponding loading control.

### Immunofluorescent Microscopy

Double immunofluorescent staining was performed for Nix to detect its expression in neurons. The total coronal sections with temporal base tissues were placed in an oven at 70°C for 60 min before staining. Then, xylenes and graded ethanol solutions to water were used to rehydrate for the sections. After antigen repair, the sections were then incubated with primary antibodies for Nix and NeuN overnight at 4°C. Appropriate secondary antibodies were added for 60 min at 37°C. Subsequently, sections were protected with cover slips with antifading mounting medium containing 4,6-diamino-2-phenyl indole (DAPI, Southern Biotech, Birmingham, AL, United States). Images were captured by using a fluorescent microscope (Nikon, Tokyo, Japan).

### Neurological Scoring

At 24 h after the establishment of the SAH model, rats in the second part of the experiment were scored for neurological function using the composite Garcia neuroscore (Krafft et al., 2014). This composite assessment consisted of seven independent sub-tests, including spontaneous activity (SA), axial sensation (AS), vibrissae proprioception (VP), symmetry of limb movement (LS), lateral turning (LT), forelimb outstretching (FO), and climbing (CL). The final neurological score of each animal was obtained by the sum of the scores of all sub-tests. The score was inversely proportional to the degree of neurological impairment in rats, with a minimum score of 3 points and a maximum score of 21 points (Table 1).

**TABLE 1:** Composite Garcia neuroscore.

**Category**	**Behavior**	**Score**
LS	Rat approached at least 3 walls of the cage	3
	Rat approached less than 3 walls	2
	Rat barely moved	1
	Rat did not move at all	0
AS	Rat was equally startled on both sides	3
	Asymmetric response	2
	Missing response on one side	1
VP	Rat equally turned head on both sides	3
	Asymmetric response	2
	Missing respond on one side	1
LS	All limbs were extended symmetrically	3
	Asymmetric extension	2
	Limbs on one side showed minimal movement	1
	Hemiplegia (no limb movement)	0
LT	Animal turns at least 45 degrees on both sides	3
	Animal turns equally to both sides but less than 45 degrees	2
	Unequal turning	1
	No turning at all	0
FO	Forelimbs were equally outstretched and the animal walked symmetrically on forepaws	3
	Asymmetric outstretch and impaired forepaw walking	2
	Minimal movement of one forelimb	1
	Hemiplegia (no limb movement)	0
CL	Rat climbed to the top of the surface	3
	Asymmetric or impaired climbing	2
	Animal failed to climb or showed tendency of circling	1

### Morris Water Maze (MWM)

The MWM was used to assess the learning and cognitive abilities of rats (Zhang et al., 2018). The circular pool had a diameter of 2 m and a height of 0.75 m, which was filled with water at a height of 0.4 m. The water temperature was maintained at 25 ± 2°C, and the water was blackened with an appropriate amount of non-toxic black edible pigment. The circular pool was divided into four quadrants equally, and a circular target platform with a diameter of 10 cm was concealed in a central portion of one quadrant, with the platform height 2 cm below the horizontal plane. During the training phase, the rats were randomly placed in the four quadrants to find the target platform for a time limit of 60 s. If the target platform was not found within 60 s, the rats were guided to climb the platform and were kept on the platform for 10 s to consolidate their spatial learning and memory. The SAH model was performed on the sixth day after five consecutive days of training. Over the next 4 days of testing, the rats were randomly placed in the four quadrants to find the target platform for a time limit of 60 s. The escape latency (EL) was the time spent by rats from the starting point to climbing on the target platform. The escape latency and swimming distance were recorded as indicators of the learning and cognitive abilities of rats.

### TUNEL Staining

TUNEL staining was used to assay cell apoptosis in brain tissue according to the manufacturer’s protocol (Roche, Switzerland). Briefly, paraffin sections were placed in an oven at 70°C for 1 h before staining. After dewaxing and antigen repair, the sections were then incubated in 0.1% Triton X-100 for 10 min, which were subsequently incubated with a TUNEL reaction mixture for 1 h at 37°C. Thereafter, the sections were blocked with 5% bovine serum albumin (BSA; Biosharp, China) for 30 min at 37°C and were then incubated with a NeuN primary antibody overnight at 4°C. The diluted secondary antibody was incubated for about 60 min at 37°C. Nuclei were stained with DAPI (Southern Biotech, Birmingham, AL, United States). Finally, the sections were observed through a fluorescent microscope (Nikon, Tokyo, Japan). The TUNEL-positive cells were counted by an observer who was blind to the experimental groups.

### FJB Staining

FJB is a type of polyanion fluorescein derivative that can bind to degenerating neurons sensitively and specifically. Paraffin sections were placed in an oven at 70°C for 1 h before staining. Then, xylenes and graded ethanol solutions to water were used to rehydrate for the sections. The slices were incubated in 0.06% K permanganate for 15 min, then rinsed in deionized water and then immersed in fluoro-jade working solution (0.1% acetic acid) for 30 min. The slices were then baked at 50–60°C for 15 min until the slices were completely dry. Slices were made transparent via incubation in xylene for 2 min and were then sealed with a mounting medium (DPX, Sigma-Aldrich, MO, United States). The FJB-positive cells were counted by an observer who was blind to the experimental groups.

### Statistical Analysis

GraphPad Prism 6 was used for all of the statistical analysis. Neurological scoring is presented as the median and interquartile range, they are analyzed with the one-way ANOVA since the data of each group passed the tests of normality and equal variance. All of the other data are expressed as the Mean ± Standard Deviation (SD). Data groups (two groups) with normal distribution were compared using the two-sided unpaired Student test. Differences in means among multiple groups were analyzed using one-way ANOVA or repeated measures two-way ANOVA followed by the Bonferroni/Dunn *post hoc* test. *p* < 0.05 was considered to be statistically significant.

## Results

### Severity of SAH and Mortality

The severity of SAH was blindly measured by SAH grade scale after euthanasia as above describe. The scores of SAH mean between different SAH groups has no statistical difference. As shown in Table 2, six rats were excluded because of low-grade SAH in experiment 1, and there were a total of seven rats excluded in experiment 2. Mortality rates in the first experiment: Sham 0% (0 of 12); SAH (3, 6, 12, 24, 48, 72 h, 7 days) 32.8% (41/125). Mortality rates in the second experiment: Sham 0% (0 of 18); SAH 30.8% (8 of 26); SAH + Vec 33.3% (9 of 27); SAH + over-Nix 28% (7 of 25); SAH + Ctr siRNA 35.7% (10 of 28); SAH + Nix siRNA 40% (12 of 30). There was no statistical difference among all groups (Table 2).

**TABLE 2:** Severity of SAH and mortality.

**Groups**	**Excluded**	**Mortality**
**Experiment 1**		
Sham	0	0%(0/12)
SAH (3, 6 12, 24, 48, 72 h, 7 days)	6	32.8%(41/125)
**Experiment 2**		
Sham	0	0%(0/18)
SAH	1	30.8%(8/26)
SAH + Vec	2	33.3%(9/27)
SAH + over-Nix	1	28%(7/25)
SAH + Ctr siRNA	1	35.7%(10/28)
SAH + Nix siRNA	2	40%(12/30)

### The Expression of Nix at Various Time Points in Brain Tissue of Rats Following SAH

**FIGURE 3:**
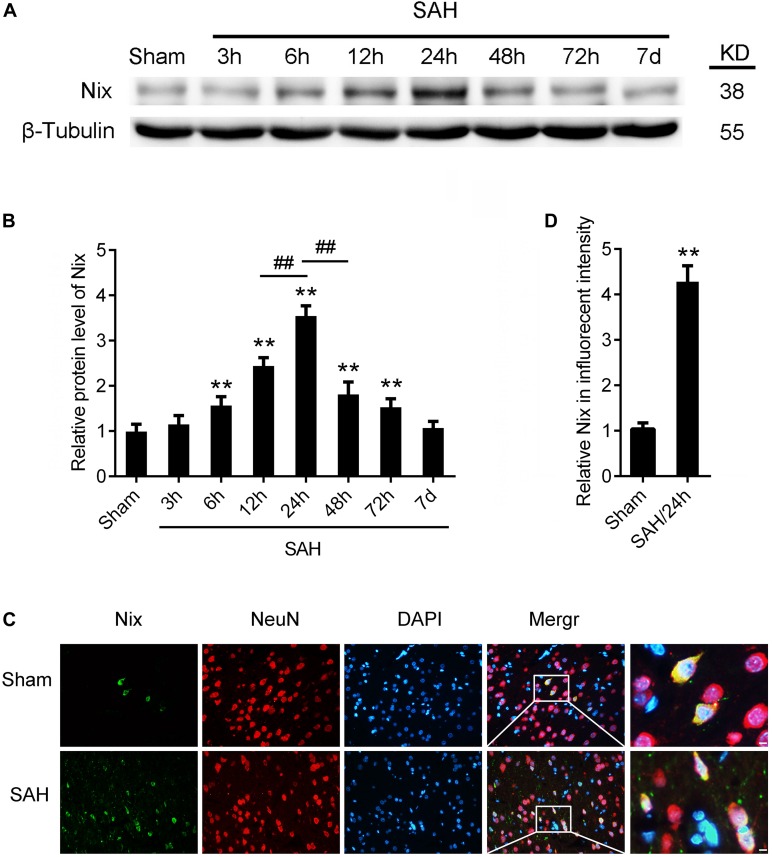
The expression of Nix in brain tissue after SAH. **(A,B)** Western blot showed that the protein level of Nix at 3, 6, 12, 24, 48, and 72 h, as well as 7 days, after SAH in brain tissues. Data represent the mean ± SD; **p < 0.01 vs. Sham group; ^##^*p* < 0.01 vs. SAH 12 h group, ^##^*p* < 0.01 vs. SAH 48 h group; *n* = 6. **(C,D)** Double immunofluorescence for Nix (green) and NeuN (red) counterstained with DAPI (blue) was performed. Representative images of the Sham group and SAH (24 h) group were shown. Data represent the mean ± SD. (Scale bar = 20 μm; **p < 0.01 vs. Sham group, *n* = 6).

In the first experiment, the expression levels of Nix in brain tissues were measured via Western blot analysis in the Sham group and SAH groups at 3, 6, 12, 24, 48, and 72 h, as well as 7 days, following SAH. As shown in Figures 3A,B, the protein level of Nix was relatively low in the Sham group. After induction of SAH, the expression of Nix in brain tissue was remarkably augmented at 6 h and peaked at 24 h post-SAH, which was significantly higher in the 24 h group compared to that of the 12 and 48 h groups. The decreased of expression of Nix continued until at least 48 h post-SAH, but there was no significant difference by 7 days post-SAH compared with the Sham group. According to these results, 24 h post-SAH was selected as the time point for further investigation.

### The Expression of Nix in Neurons of Rats Following SAH

To further confirm the expression of Nix in brain tissues, the double immunofluorescence staining was used to verify the expression of Nix in neurons after SAH. As shown in Figures 3C,D, in the temporal base tissues of sections, the immunofluorescence staining of Nix in the Sham group was weak. However, the Nix immunofluorescence was strongly enhanced in neurons at 24 h after SAH. These changes indicated a significant increase in the expression of Nix in neurons at 24 h after SAH compared with that in the Sham group, which was consistent with our results of Western blot.

### Effect of Nix on the Mitochondrial Marker Protein (TOMM20) Following SAH

**FIGURE 4:**
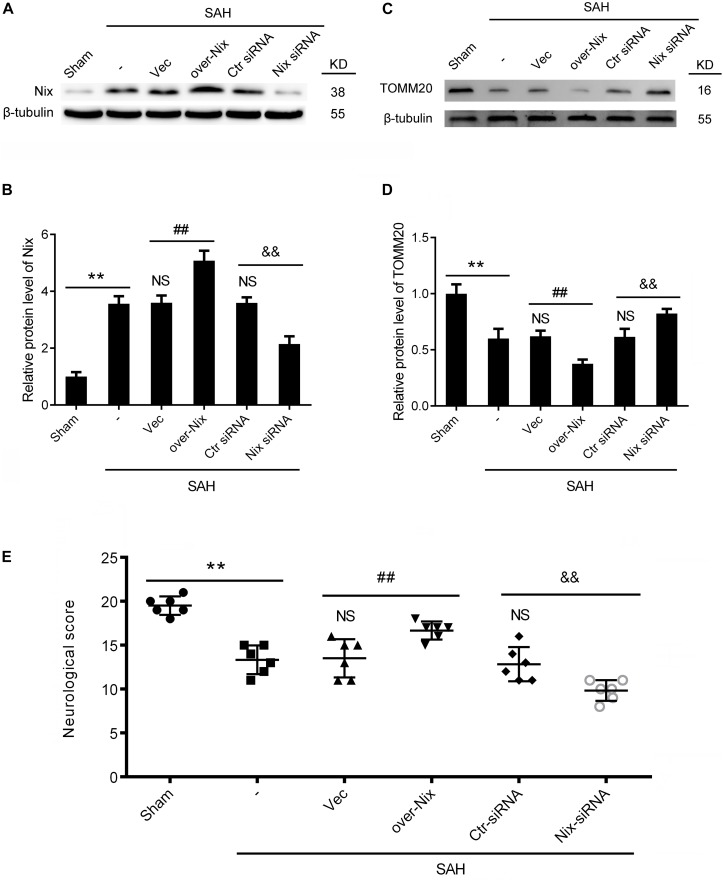
Effects of Nix on EBI at 24 h after SAH. **(A–D)** Western blot showed the protein levels of Nix and the mitochondrial marker protein, TOMM20, in the Sham, SAH, SAH + Vec, SAH + over-Nix, SAH + Ctr siRNA, and SAH + Nix siRNA groups at 24 h after SAH. Data represent the mean ± SD; **p < 0.01 vs. Sham group; ^NS^*p* > 0.05 vs. SAH group; ^##^*p* < 0.01 vs. SAH + Vec group; ^&&^*p* < 0.01 vs. SAH + Ctr siRNA group; *n* = 6. **(E)** Composite Garcia neuroscore; **p < 0.01 vs. Sham group;^NS^*p* > 0.05 vs. SAH group; ^##^*p* < 0.01 vs. SAH + Vec group; ^&&^*p* < 0.01 vs. SAH + Ctr siRNA group; *n* = 6.

The Nix overexpression plasmids and Nix siRNAs were administered separately through intracerebroventricular injections at 24 h before induction of SAH to bidirectionally regulate the expression of the Nix. The effects of bidirectionally regulation of Nix were first ascertained via Western blot. As shown in Figures 4A,B, the protein level of Nix in the SAH + over-Nix group was significantly higher than that in the SAH + Vec group. In contrast, the protein level of Nix in the SAH + Nix siRNA group was significantly suppressed after SAH compared with that in the SAH + Ctr siRNA group. Meanwhile, as shown in Figures 4C,D, administering a Nix overexpression plasmid remarkably depressed the expression of the mitochondrial marker protein, TOMM20, which was evidently augmented in the SAH + Nix siRNA group.

### Cognitive Behavior and Neurological Impairment in Rats Following SAH

**FIGURE 5:**
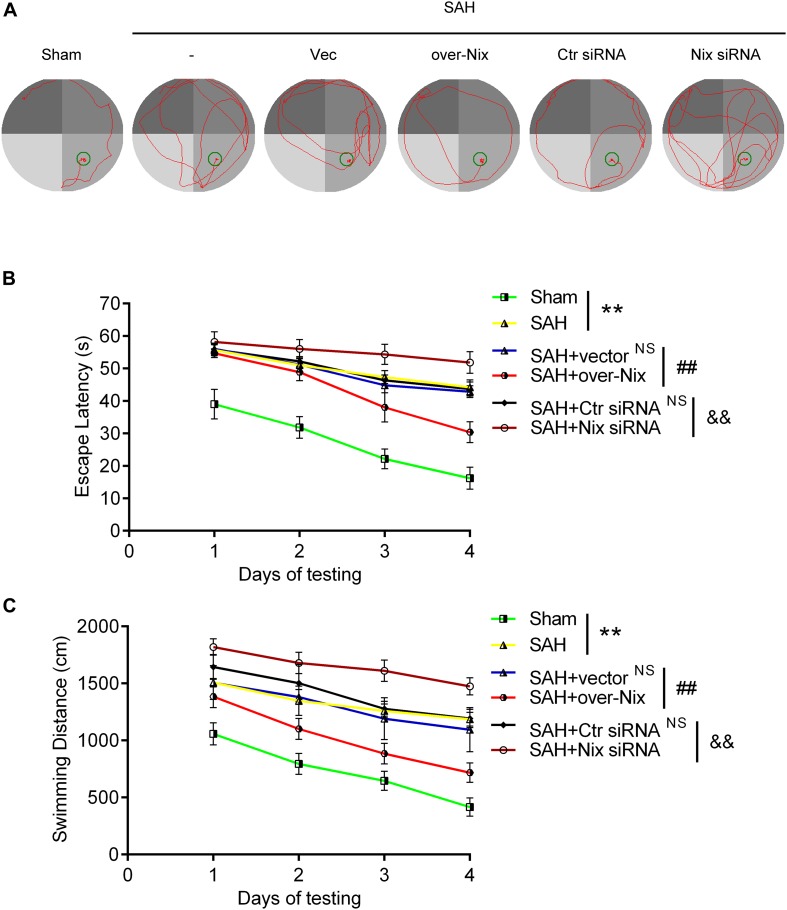
Cognitive behavioral impairment of rats were detected via the Morris water maze. **(A)** Representative tracing images from the Morris water maze test in the Sham, SAH, SAH + Vec, SAH + over-Nix, SAH + Ctr siRNA, and SAH + Nix siRNA groups. **(B,C)** Escape latencies and swimming distance over 4 days. Data represent the mean ± SD; **p < 0.01 vs. Sham group; ^NS^*p* > 0.05 vs. SAH group; ^##^*p* < 0.01 vs. SAH + Vec group; ^&&^*p* < 0.01 vs. SAH + Ctr siRNA group; *n* = 6.

To further investigate the effect of Nix on the EBI following SAH in rats, we used the composite Garcia neuroscore to assess the neurological impairment, whereas cognitive function was evaluated via the Morris water maze. As shown in Figures 4E, 5, remarkable neurological impairment was observed in the SAH group compared that of the Sham group. Intracerebroventricular injection of a Nix overexpression plasmid significantly ameliorated SAH-induced neurological impairment in rats. On the contrary, neurobehavioral deficits were significantly exacerbated after administering the Nix siRNA. Meanwhile, one representative tracing image of the fourth day of testing phase from each group was displayed in Figure 5A. There were no significant differences in swimming speed among the experimental groups, which suggested that swimming ability did not grossly differ between the groups. As a result, rats in all SAH groups showed severe impairments in cognitive behavior compared with those in the Sham group. The SAH + over-Nix group exhibited significantly shorter swimming distance than the SAH + Vec group began with the second day. Similarly, in the escape latency, the SAH + over-Nix group exhibited significantly compared with the SAH + Vec group on the third and fourth days. Contrarily, the swimming distance of the SAH + Nix siRNA group increased significantly by day one compared with the SAH + Ctr siRNA group. Moreover, there was significant difference between the SAH + Nix siRNA group and SAH + Ctr siRNA group by day two.

### Neuronal Apoptosis and Neurodegeneration in Brain Tissue Following SAH

**FIGURE 6:**
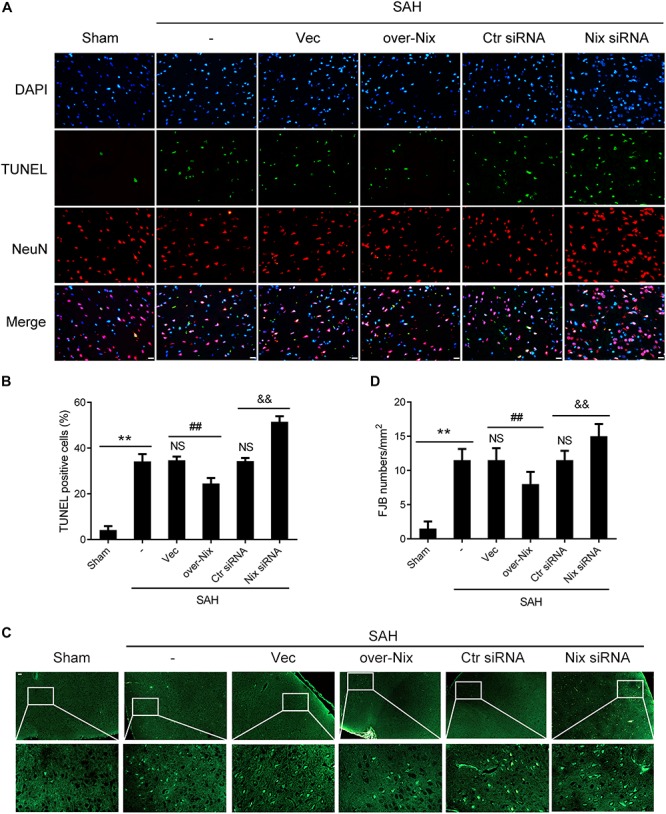
Effects of Nix on the neuronal apoptosis and degeneration at 24 h after SAH. **(A,B)** TUNEL staining showed apoptotic cells in the Sham, SAH, SAH + Vec, SAH + over-Nix, SAH + Ctr siRNA, and SAH + Nix siRNA groups. Double-immunofluorescent staining was performed with TUNEL (green) and a neuronal marker (NeuN, red), and nuclei were fluorescently labeled with DAPI (blue), Scale bar = 20 μm; **p < 0.01 vs. Sham group; ^NS^*p* > 0.05 vs. SAH group; ^##^*p* < 0.01 vs. SAH + Vec group; ^&&^*p* < 0.01 vs. SAH + Ctr siRNA group; *n* = 6. **(C,D)** FJB fluorescence staining (green) showing neuronal degeneration. Scale bar = 20 μm. **p < 0.01 vs. Sham group; ^NS^*p* > 0.05 vs. SAH group; ^##^*p* < 0.01 vs. SAH + Vec group; ^&&^*p* < 0.01 vs. SAH + Ctr siRNA group; *n* = 6.

As shown in Figure 6, compared with those in the Sham group, histological examination indicated that the numbers of TUNEL-positive neurons and FJB-positive neurons were significantly higher in the SAH group. Remarkably, compared with that in the SAH + Vec group, treatment with a Nix overexpression plasmid markedly restored the total number of TUNEL and NeuN double-labeled cells in brain tissues, as well as number of FJB-positive cells. On the contrary, the total numbers of TUNEL/NeuN double-labeled cells was significantly increased after Nix siRNA treatment compared with that in the SAH + Ctr siRNA group. Similarly, the SAH + Nix siRNA group revealed that a significant increase in the number of FJB-positive cells compared with that of the SAH + Ctr siRNA group.

## Discussion

In this study, we performed a controllable autologous blood injection model in adult rats to mimic SAH *in vivo*. EBI is an important factor leading to the deterioration of SAH patients and refers to a series of pathophysiological processes within 72 h after SAH, including ER stress, organelle injury, cell death, destruction of the BBB, cerebral vasospasms, edema, and oxidative stress (Dou et al., 2017). Most previous studies on EBI have been concentrated on the first 72 h after SAH (Liu et al., 2018). Similarly, in this experiment, we also focused on the changes of Nix expression within 72 h after SAH. At the same time, in order to further observe the expression of Nix at the later stages of SAH, we extended our observed time points to 7 days after SAH. We found that the protein level of Nix in temporal basal brain tissue of rats in the Sham group was relatively low, but increased gradually and reached a peak at 24 h after SAH, after which it decreased gradually. Therefore, we focused at 24 h after SAH to further explore the role of Nix in the second experiment of our present study. Previous studies have shown that the expression of Nix in brain tissue is mainly located in neurons compared to that in astrocytes and oligodendrocytes (Prabhakaran et al., 2007; Rui et al., 2013; Yuan et al., 2017). In order to further verify the expression of Nix in neurons, we co-stained Nix and the neuron marker, NeuN, in brain tissues of rats in the Sham group and 24 h SAH group via double-immunofluorescent staining. The results also confirmed that the expression of Nix after SAH was significantly higher than that in the Sham group, which is consistent with the results of previous studies.

In our second experiment, the Nix levels were increased and suppressed via intracerebroventricular injection of Nix-specific overexpression plasmids and siRNAs, respectively. Firstly, we detected the protein level of Nix by Western blot, ensuring successful transfection and effective manipulation of Nix levels. The amount of mitochondria can be directly reflected through the protein level of TOMM20, a marker protein of mitochondria, which is located in the outer membrane of mitochondria. The result of Western blot revealed that the amount of mitochondria decreased significantly after Nix overexpression, while the amount of mitochondria increased significantly after inhibiting the expression of Nix, but it was still lower than that of the Sham group. Then, we assessed the cognitive abilities in each group of rats via the MWM. Overexpression of Nix significantly ameliorated SAH-induced cognitive impairment, whereas these impairments were further exacerbated via inhibiting Nix expression via siRNA knockdown. Subsequently, we used the Garcia neuroscore to assess the neurological dysfunction of rats in each group, and these results were consistent with our MWM results. Finally, the apoptosis and degeneration of neurons were assayed via TUNEL and FJB staining respectively. The results indicated that overexpression of Nix reduced the numbers of both TUNEL-positive neurons and FJB-positive neurons, while Nix siRNA treatments increased these numbers.

Previous studies have shown that the mitochondrial injury and functional disturbances are an important cause of the death of the neurons following SAH (Chen et al., 2014; Jiang et al., 2019). Mitophagy is a process in which cells selectively clear mitochondria through autophagy (Wu et al., 2016). During mitophagy, damaged mitochondria are specifically recognized and wrapped in autophagosomes and are then fused with lysosomes, thereby being degraded in order to regulate the amount of mitochondria based on current metabolic needs (Band et al., 2009). Additionally, mitophagy is also an important way to achieve the quality control of mitochondria by eliminating damaged mitochondria, which plays a significant role in maintaining cellular homeostasis (Rehm et al., 2009). In recent years, many studies have shown that Nix is expressed in almost all of the outer membranes of mitochondria and can mediate mitophagy (Esteban-Martinez and Boya, 2018). In ischemic stroke, it has been confirmed that Nix protects against brain injury by mediating mitophagy (Sandoval et al., 2008; Yuan et al., 2017).

Additionally, although the amount of mitochondria in the SAH + Vec group was higher than that in the SAH + over-Nix group, a larger proportion of these may have been injured mitochondria, which are known to induce secondary injury to cells and organisms (Srivastava, 2017). It has been shown that accelerating the removal of injured mitochondria after the overexpression of Nix ameliorates brain injury (Campello et al., 2014). However, the injured mitochondria in cells cannot be cleared in time when the protein level of Nix is inhibited (Palikaras and Tavernarakis, 2014). This results in a series of secondary injury that exacerbates SAH-induced brain injury after SAH (Redmann et al., 2014; Fivenson et al., 2017). Thus, Nix regulation of mitochondria may not only be for quantitative control, but also quality control, both of which are indispensable to maintain the stability of cells and organisms (Kurihara et al., 2012).

Recent studies have found that Nix plays an important role in mediating mitophagy (Xiang et al., 2017). However, the specific mechanism for this phenomenon remains unclear. At present, there are mainly three hypotheses for the possible mechanism of Nix-mediated mitophagy. Firstly, most of the known autophagic receptors mediate autophagy by interacting with members of the Atg8 family (Wei et al., 2015). Nix has two LC3-interaction region (LIR) motifs, which comprise the momentous structural domain that binds the Atg8 ortholog, LC3, and the LC3 homolog, GABA receptor-associated protein (GABARAP) in mammalian cells (Rogov et al., 2017). Nix, as a protein of autophagy receptor, recruits members of the Atg8 family to the injured mitochondria through its own LIR motifs, thereby resulting in the removal of the mitochondria (Schwarten et al., 2009). Secondly, the PINK1/Parkin pathway is a significant pathway mediating mitophagy, and the activation of this pathway is associated with a decrease in the membrane potential of mitochondria (Zhao et al., 2018). Some studies have shown that high expression of Nix can induce a decrease of the mitochondrial membrane potential, and then activate the PINK1/Parkin pathway to co-mediated mitophagy (McLelland et al., 2014; Gao et al., 2015). Additionally, Nix can be ubiquitinated via Parkin, which promotes its recognition by autophagic receptors, ultimately leading to the autophagic clearance of mitochondrial (Kane et al., 2014). Thirdly, Beclin-1 is an important protein that is involved in the generation of autophagosomes. Bcl-2 can bind to Beclin-1 and inhibit mitophagy (Oberstein et al., 2007). BH3-only protein or its analogs can release free Beclin-1 to induce mitophagy by competing with Beclin-1 to bind Bcl-2 or Bcl-XL (Feng et al., 2007). Therefore, Nix, as a BH3-only protein, may also mediate mitophagy through the above mechanism.

Also, our present study had some limitations. First, we did not design *in vitro* experiments to explore the role of Nix after SAH. Secondly, mitophagy, a vital means for cells to control the quantity and quality of mitochondria, plays a significant role in maintaining the stability of cells. As an important protein for mediating autophagy, Nix takes part in many important physiological and pathological processes, but its specific mechanism is not completely understood. Similarly, this experiment was also unable to clarify the specific mechanism of Nix-mediated mitophagy. Finally, this study did not elucidate how Nix specifically recognizes injured mitochondria after SAH to confer selective mitophagy. In summary, in the present study, the expression of Nix was found to increase after SAH in rats and to play a neuroprotective role during the EBI after SAH. As stated at the beginning of this article, SAH remains a serious disease with the high disability and mortality rates. In this study, we discovered that Nix could be a new target for the treatment of SAH, which had a potential clinical translation value. However, it is now difficult to achieve the translation from bench to bedside because of the undefined mechanisms and species difference. Importantly, future studies that reveal the precise mechanisms of Nix-mediated mitophagy may provide novel therapeutic approaches for stroke patients.

## Data Availability Statement

The datasets generated for this study are available on request to the corresponding author.

## Ethics Statement

The animal study was reviewed and approved by Institutional Animal Care Committee of the First Affiliated Hospital of Soochow University.

## Author Contributions

JZ and GY conducted the experiments and drafted the manuscript. TL, PP, XL, and HL contributed to the experiment design and statistical analysis. GC, ZW, and HS designed the experiments and revised the manuscript.

## Conflict of Interest

The authors declare that the research was conducted in the absence of any commercial or financial relationships that could be construed as a potential conflict of interest.

## Supplementary Material

DATA SHEET S1 Original image files for the blots.
