# Supplementary figures and images for: Nix Plays a Neuroprotective Role in Early Brain Injury After Experimental Subarachnoid Hemorrhage in Rats
Source: Front Neurosci. 2020 Mar 24;14:245. doi: 10.3389/fnins.2020.00245 (PMC7108665; doi:10.3389/fnins.2020.00245)

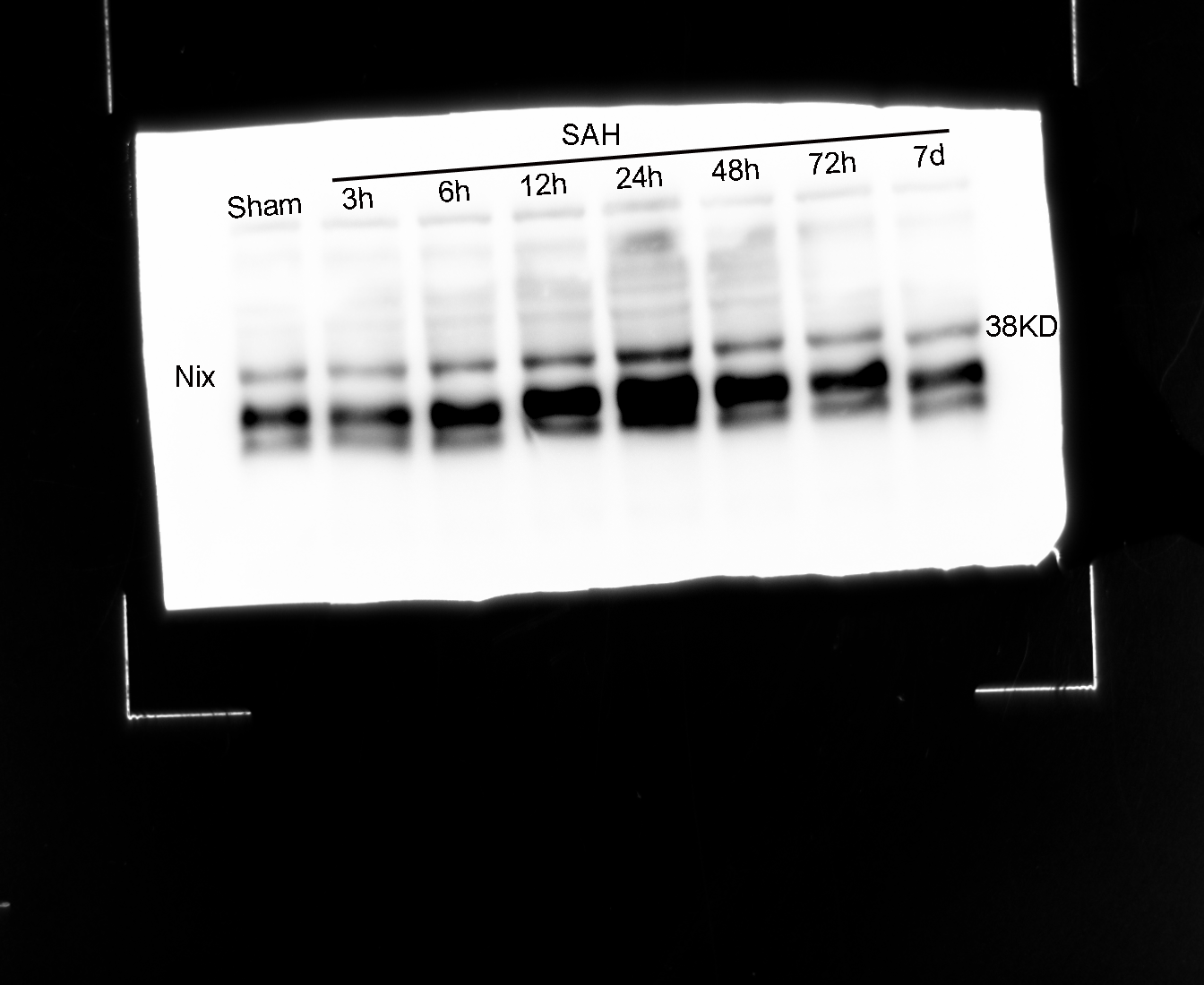

Supplement: DATA SHEET S1 — Original image files for the blots. [file Data_Sheet_1.ZIP › Experiment 1 Nix.tif]

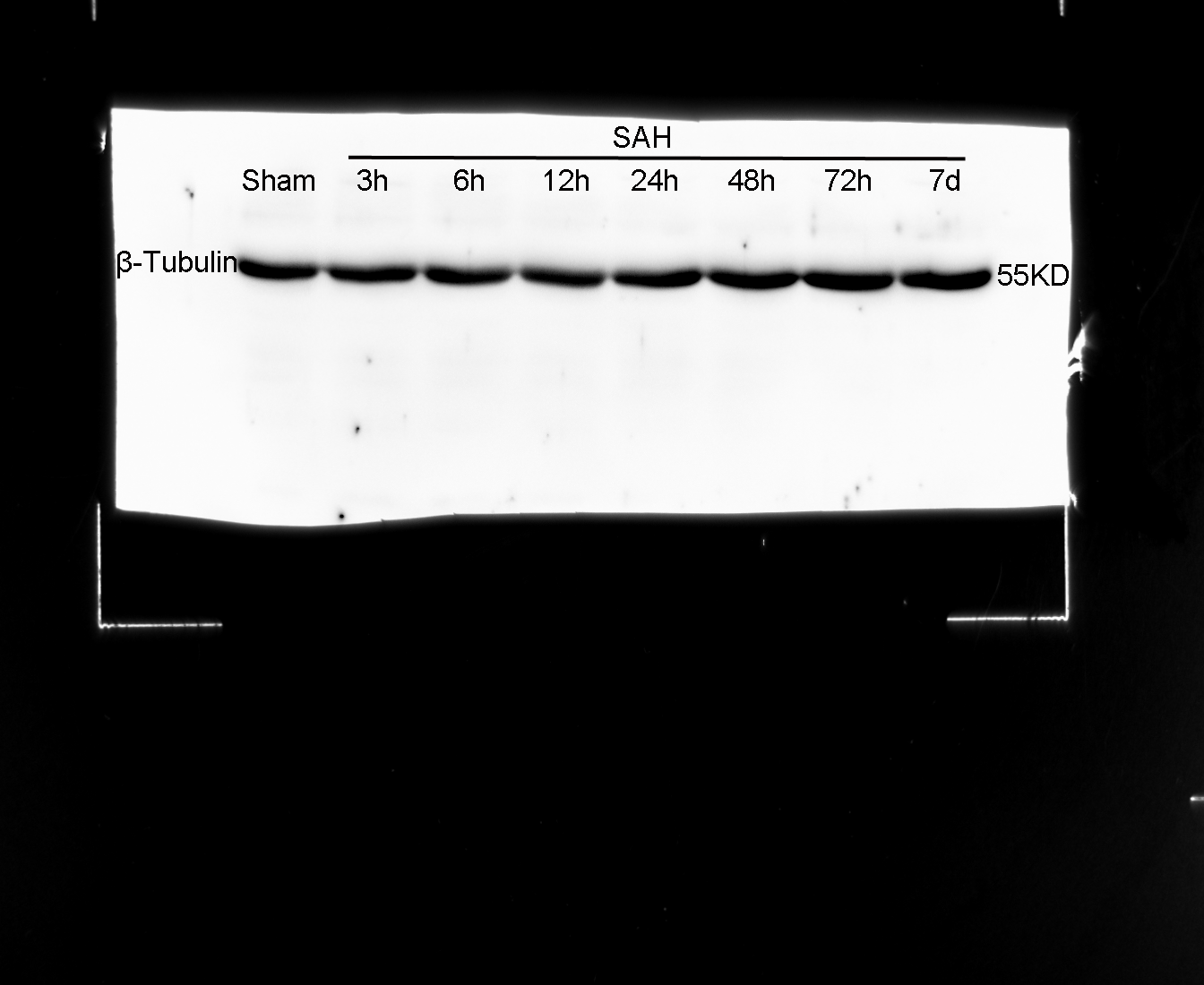

Supplement: DATA SHEET S1 — Original image files for the blots. [file Data_Sheet_1.ZIP › Experiment 1 β-Tubulin (Nix).tif]

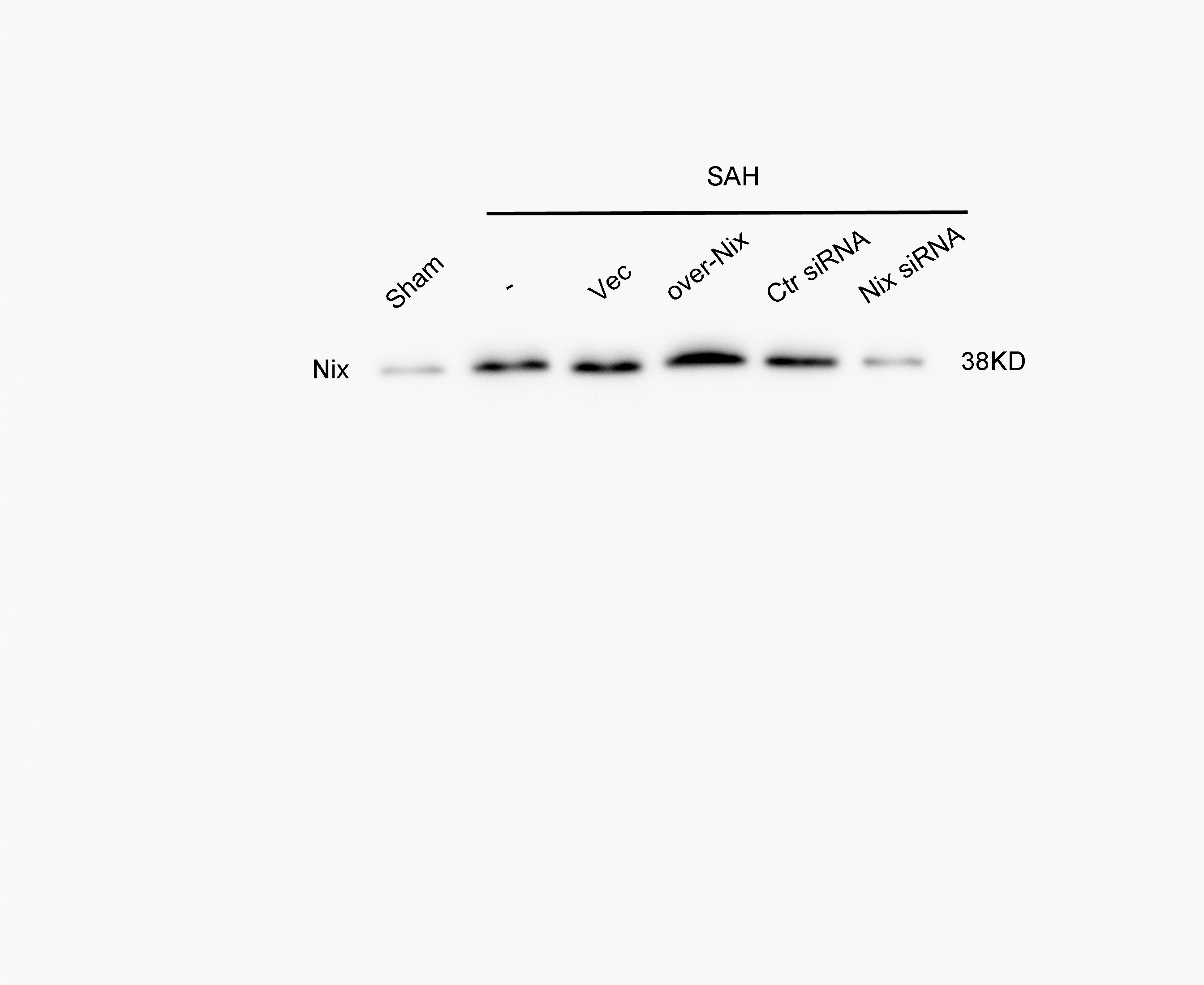

Supplement: DATA SHEET S1 — Original image files for the blots. [file Data_Sheet_1.ZIP › Experiment 2 Nix.tif]

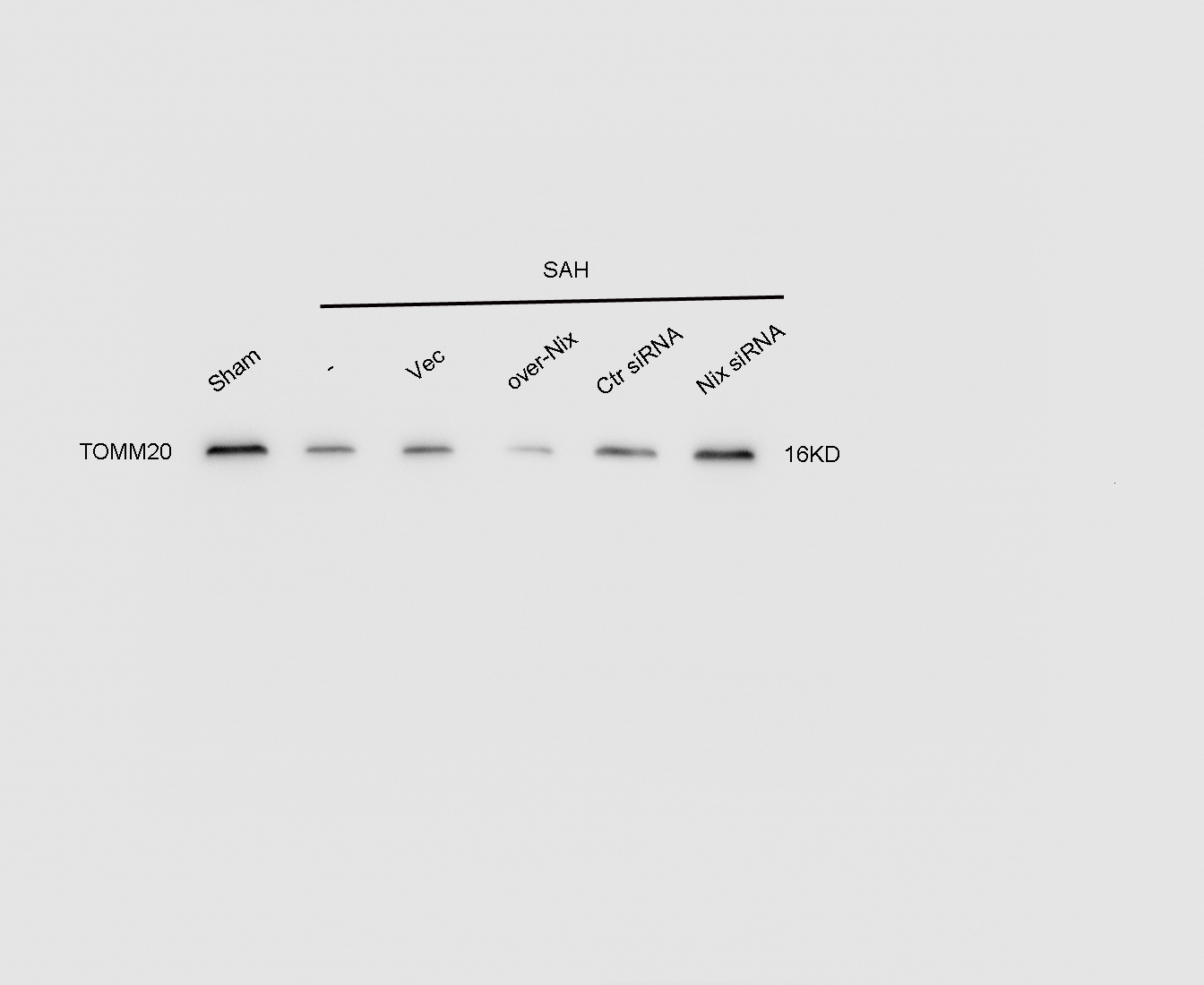

Supplement: DATA SHEET S1 — Original image files for the blots. [file Data_Sheet_1.ZIP › Experiment 2 TOMM20.tif]

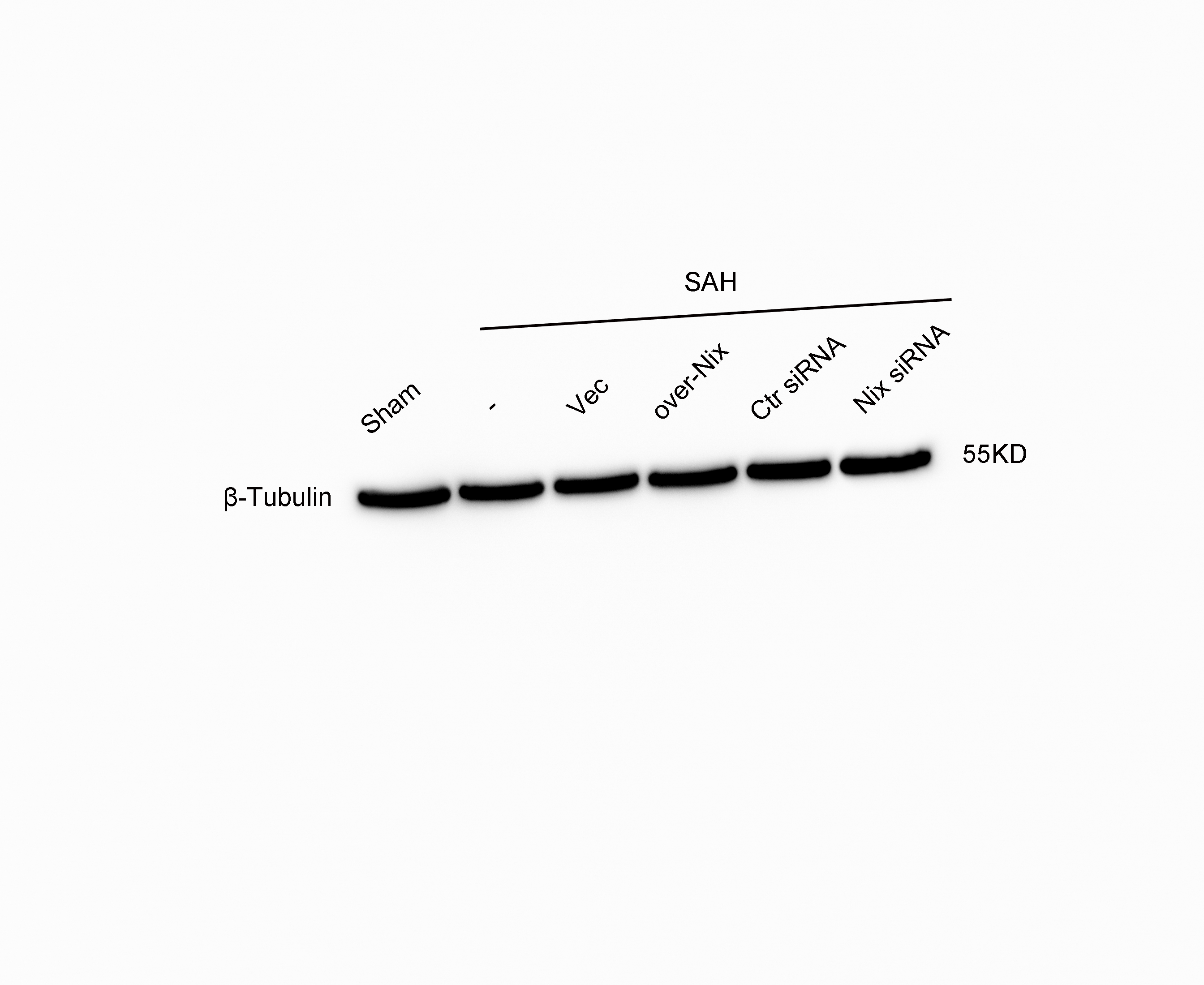

Supplement: DATA SHEET S1 — Original image files for the blots. [file Data_Sheet_1.ZIP › Experiment 2 β-Tubulin (Nix).tif]

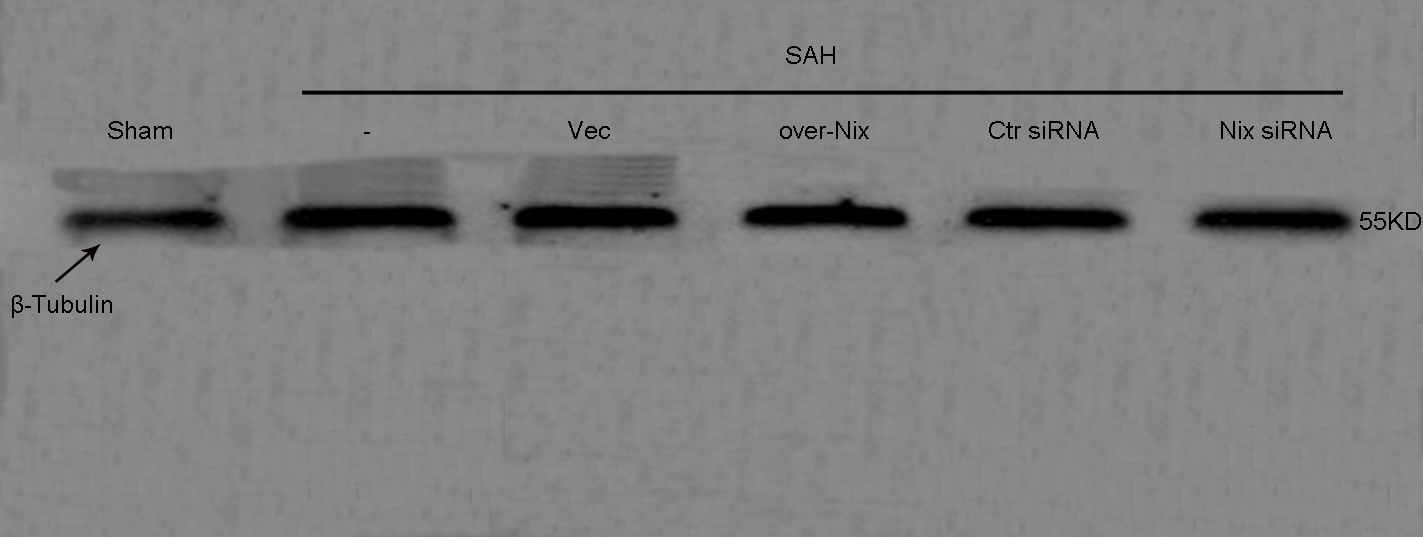

Supplement: DATA SHEET S1 — Original image files for the blots. [file Data_Sheet_1.ZIP › Experiment 2 β-Tubulin (TOMM20).tif]
